# Overexpression of *OsCM* alleviates BLB stress via phytohormonal accumulation and transcriptional modulation of defense-related genes in *Oryza sativa*

**DOI:** 10.1038/s41598-020-76675-1

**Published:** 2020-11-11

**Authors:** Rahmatullah Jan, Muhammad Aaqil Khan, Sajjad Asaf, In-Jung Lee, Jong-Sup Bae, Kyung-Min Kim

**Affiliations:** 1 Division of Plant Biosciences, School of Applied Biosciences, College of Agriculture and Life Science, Kyungpook National University, 80 Dahak-ro, Buk-gu, Daegu, 41566 Republic of Korea; 2 Natural and Medical Science Research Center, University of Nizwa, 616 Nizwa, Oman; 3 College of Pharmacy, CMRI, Research Institute of Pharmaceutical Sciences, BK21 Plus KNU Multi-Omics Based Creative Drug Research Team, Kyungpook National University, Daegu, 41566 Republic of Korea

**Keywords:** Cell biology, Developmental biology, Genetics, Molecular biology, Plant sciences

## Abstract

*Xanthomonas oryzae* is a serious pathogen causing bacterial leaf blight (BLB) disease in rice, markedly reducing its yield. In this study, the rice chorismate mutase (*OsCM*) gene was overexpressed in a bacterial leaf blight-susceptible rice line to investigate the functional role of *OsCM* in response to bacterial leaf blight stress. We reported that overexpression of *OsCM* altered the downstream pathway of aromatic amino acids, mitigating pathogen stress by altering stress-responsive genes and hormonal accumulation. Phenotypic evaluation showed that the lesion length in the transgenic line was significantly lesser than that in the wild-type, suggesting greater resistance in the transgenic line. Further analysis revealed that *OsCM* expression induced phenylalanine accumulation and suppressed tyrosine accumulation in response to bacterial leaf blight stress. Furthermore, bacterial leaf blight stress induced genes downstream of the phenylpropanoid pathway in conjunction with *OsCM*, suggesting that the phenylpropanoid pathway is dependent on *OsCM* gene expression. We reported high SA and low JA accumulation in response to bacterial leaf blight stress in the transgenic line. This higher SA accumulation suggested that SA induces immune responses by functioning as a promoter of nonexpresser pathogenesis-related genes 1 (*NPR1*) transcriptional regulation. *Xa7* expression was induced with increase in nonexpresser pathogenesis-related genes 1, which is thought to be responsible for *Xa7* expression, which is responsible for mitigating bacterial leaf blight stress.

## Introduction

The yield of rice, a staple food for about 50% the world’s population, is decreased due to biotic and abiotic stresses. Among the biotic stresses, bacterial leaf blight (BLB) is one of the most severe and common rice diseases in most Asian countries; it is caused by *Xanthomonas oryzae* pv. *oryza* (Xoo) and results in considerable yield loss each year^1^. Xoo can be more successfully controlled by the development of transgenic resistant varieties of rice, usually through single-gene resistance^2^. Generally, the exposure of plants to pathogens enhances the induction of genes involved in the shikimate pathway and aromatic amino acids (AAAs), such as phenylalanine (Phe), tyrosine (Tyr), and tryptophan (Trp)^3^. Pathogenic attack stimulates the plant cell wall and releases oligogalacturonides, which in turn stimulate the expression of various genes encoding enzymes of the shikimate pathway and AAAs as well as those encoding secondary metabolites derived from Phe and Tyr^4^. Bacterial pathogens redirect normal host metabolism by delivering a constellation of type III effector proteins to enhance pathogen multiplication and nutrition^5,6^. However, genes associated with AAAs have been significantly modified to cope with bacterial challenges, including cell wall alteration to control the nutrients and water passage from plants to invading bacteria^7,8^. Phe, Tyr, and Trp are the main AAA molecules in plant metabolism which are responsible for synthesis of a number of hormones, such as salicylic acid and auxin, as well as for essential secondary metabolites with many biological functions^9,10^.

Chorismate along with the synthesis of salicylic acid (SA) through isochorismate synthase (*ICS*) activity^11^ is also the main precursor and initial branch point metabolite in the synthesis of all AAAs and their derivatives. The main route of Phe and Tyr biosynthesis is initiated from the same precursor, chorismite, catalyzed by the chorismate mutase (*CM*) enzyme to produce prephenate, which is further catalyzed to arogenate by the prephenate aminotransferase (*PAT*) enzyme. Phe and Tyr are synthesized in two ways. First, prephenate is converted to phenylpyruvate and hydroxyphenylpyruvate via the prephenate dehydratase (*PDT*) and prephenate dehydrogenase (*PDH*) enzymes, respectively. Further, phenylpyruvate and hydroxyphenylpyruvate are catalyzed into Phe and Tyr, respectively, through the aromatic amino acid aminotransferase (*AAAAT*) enzyme. Alternately, prephenate synthesizes arogenate through the prephenate aminotransferase (*PAT*) enzyme, which is further catalyzed into Phe and Tyr through the arogenate dehydratase (*ADT*) and arogenate dehydrogenase (TyrA) enzymes, respectively^12–14^. Previous reports support the observation that, in phenylpyruvate containing plants, it also act as a precursor of various secondary metabolites^15^. Phe and Tyr are catabolized to produce anabolic precursors of the most important secondary metabolites through the phenylpropanoid pathway. Phenylalanine ammonia lyase (PAL) is responsible for conversion of Phe into cinnamate; however, Tyr is the directly involved in the synthesis of coumarate in the phenylpropanoid pathway by the tyrosine ammonia lyase enzyme^16^. The gene responsible for the PAL enzyme is regulated during biotic stress and under conditions that increase the need for lignin as a cell wall component^17^. The metabolites of the phenylpropanoid pathway causes toughness to cells which act as pest deterrent and disease resistant^18^. The PAL enzyme is involved in the first step of the phenylpropanoid pathway, through which the synthesis of various secondary metabolites (flavonoid pigments, lignin, antimicrobial phenolics, UV protectants, and cell wall-associated phenolics) take place against biotic, mechanical, and UV stresses, which is a definitive defense reaction to pathogenic attack^19^. Expression of the *CM* gene induces PAL activity, providing precursors for the biosynthesis of lignin and SA, which change in response to pathogen infection. SA accumulation is essential for systemic acquired resistance (SAR), removing H_2_O_2_ and suppressing the oxidative burst necessary for the HR. A report has shown that SA plays an important role in Xoo resistance in rice and promotes basal as well as hypersensitive responses during Xoo infection in rice^20^. The accumulation of SA increases under stress conditions, which translocates *NPR1* into the nucleus for SA-dependent transcriptomic changes^21^. *NPR1* is hypothesized to be a transcription factor due to its lack of a DNA binding domain and responsibility for Xoo resistance in rice, given that it is responsible for PR gene expression^22^. Furthermore, the SA and JA signaling pathways intersect at various branches because they alter biotic stress antagonistically, which was reported for the first time in tomato^23^. SA inhibits JA accumulation by suppressing JA-responsive genes, which suggests that *NPR1* is a key player in the antagonistic cross-talk of SA and JA^24^.

Despite of great importance of chorismate mutase in alteration of shikimate pathway and biosynthesis of AAAs, the role of chorismate mutase in the stress response has been mostly ignored. Although most of the researchers evaluated the function of chorismate mutase associated with the aromatic amino acids and secondary metabolites, but still it is not explored in response to biotic stress. Therefore, the present study focus on role of chorismate mutase in the alteration of the PAL pathway and biotic stress tolerance (BLB) in terms of aromatic amino acid and SA accumulation and transcriptional modulation of defense-related genes in *Oryza sativa*.

## Results

### Cloning and *OsCM-*overexpressing line development

**Figure 1:**
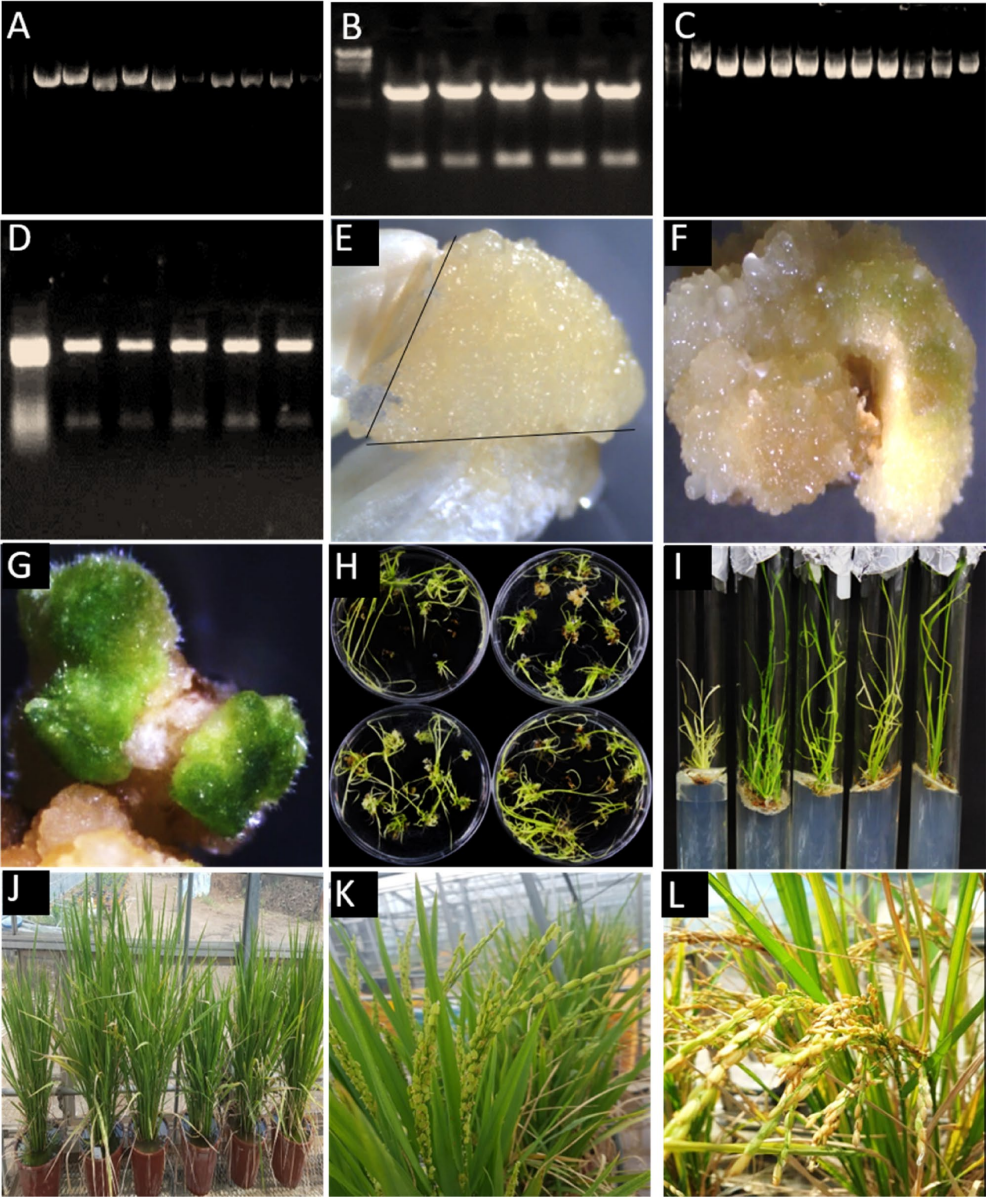
Cloning and development of *OsCM*-overexpressing line. (**A**) Cloning into *E. coli*. (**B**) Double digestion of cloned plasmid isolated from *E. coli.* (**C**) Cloning into *Agrobacterium*. (**D**) Double digestion of cloned plasmid isolated from *Agrobacterium*. (**E**–**L**) Developmental stages of transgenic line through callus culture.

The complete ORF region of *OsCM* was successfully cloned into a cloning vector and transferred to *E. coli* cells for multiplication. The isolated plasmid and double digestion with the restriction enzymes Not1 and Asc1, are shown in Fig. 1A,B. The double-digested template was further cloned successfully into an expression vector and transferred into *Agrobacterium* cells. The plasmid isolated from *Agrobacterium* was double-digested with BamH1 and Xho1 restriction enzymes (Fig. 1C,D). The *OsCM*-overexpressing rice line was developed using callus culture; its various developmental stages are described in Fig. 1E–L. The efficiency of transgenic line development was about 18% using hygromycin as a selection marker. The seeds obtained were used for further analysis.

### *OsCM* is functionally involved in BLB tolerance

**Figure 2:**
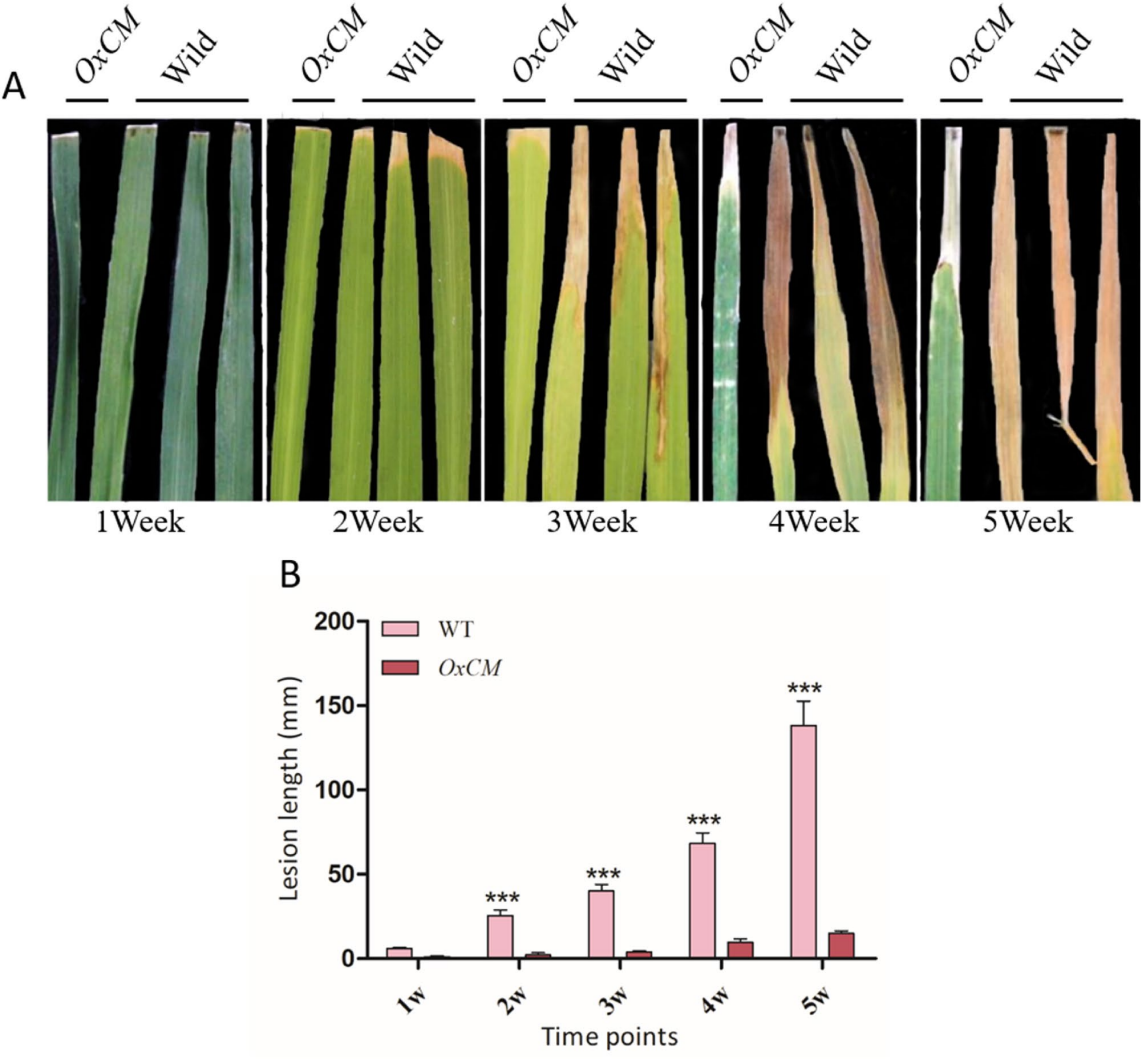
Phenotypic validation of pathogen infection. (**A**, **B**) Image and graphical representation of lesion length in response to pathogen infection, respectively. Bars represent mean ± standard deviation, while asterisks indicate a significant difference (*p*˂0.05, two-way ANOVA, Bonferroni posttest). 1w, 2w, 3w, 4w, and 5w represent time points for obtaining data (in weeks).

To evaluate the differences in morphological characteristics of *OxCM* and wild-type (WT) rice plants, using the leaf clipping method the plants were inoculated with K3a strain of Xoo. The lesion length upon BLB infection of WT as well as *OxCM* plants was measured from 1 week after inoculation of the K3a strain, for 5 consecutive weeks. The inoculation of K3a was confirmed through colony counting. The phenotypic evaluation showed that the lesion length increased markedly after each week, indicating that WT plants were highly susceptible to K3a infection (Fig. 2A). However, the lesion length of *OxCM* plants was much less throughout the five weeks, suggesting that *OxCM* expressing plant was highly tolerant of K3a.The infection of K3a in WT plants reached the severe stage and finally the leaves died after 5 weeks. The measurement of lesion length showed that the lesions increased from 6 to 138 mm from the first to the fifth week in WT plants (Fig. 2B). However, in *OxCM* plants, the lesions increased from 1.1 to 15 mm from the first to the fifth week. This shows that overexpression of *OsCM* in rice significantly (*P* < 0.001) enhanced tolerance to BLB.

### Pathogen infection enhances *OsCM* expression and phenylalanine accumulation and suppresses tyrosine

**Figure 3:**
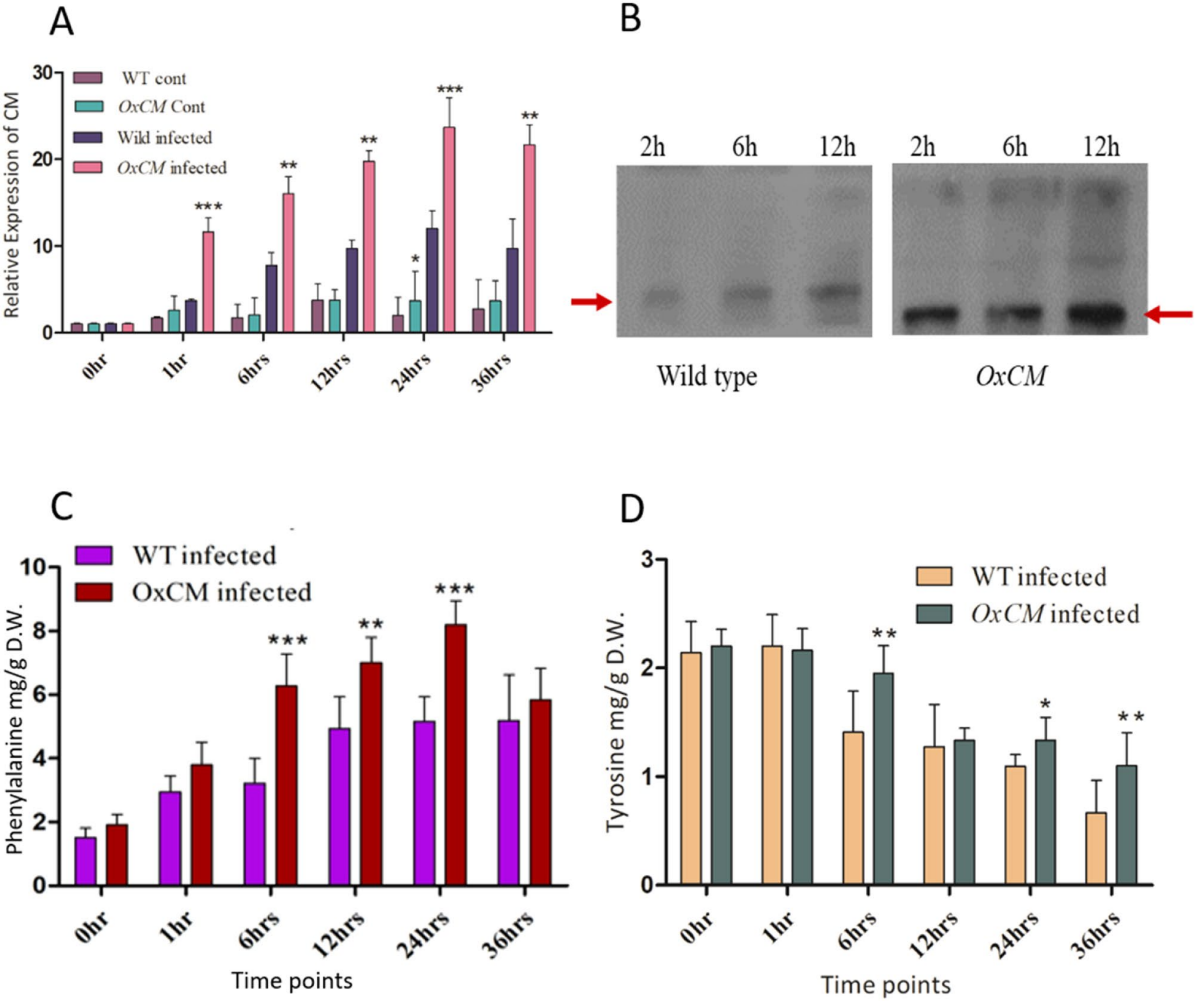
Functional expression of *OsCM* gene and Phe and Tyr accumulation. Bars represent mean ± standard deviation, while asterisks indicate a significant difference (*p *˂ 0.05, two-way ANOVA, Bonferroni post-test). 0 h, 1 h, 6 h, 12 h, 24 h, and 36 h represent time points when data were obtained (in h). WT cont refers to the WT control, *OxCM* cont refers to the CM-overexpressing control line, wild infected is the WT infected with BLB, and *OxCM* infected is the CM-overexpressing line infected with BLB. (**A**) Relative expression of the *OsCM* gene in WT and overexpressing control and infected plants. (**B**) Protein expression in the WT and *OxCM* line at 2, 6, and 24 h after infection with BLB. The provided (**B**) is cropped due to the multiple samples blotting on same X-ray film due to time and resources limitation, the original non-processed picture is given in Supplementary Figure S1. (**C**, **D**) Phenylalanine and tyrosine quantification in WT and *OxCM* infected plants.

To further investigate the biological function of *OsCM* in Phe and Tyr accumulation in stressed as well as normal conditions, we infected WT plants and *OxCM* plants and compared them with uninfected WT and *OxCM* plants. The *OsCM* gene expression in the transgenic infected line was significantly different (*P* < 0.001) from that of WT infected plants (Fig. 3A). However, the expression of *OsCM* in uninfected plants was not significantly higher than that of WT uninfected plants. We also investigated the level of *OsCM* protein expression in transgenic and WT infected plants. The results showed that *OsCM* protein was significantly expressed in the *OxCM* line as compared to the WT plants (Fig. 3B). These results show that *OsCM* is positively involved in pathogen resistance. We further investigated Phe and Tyr accumulation in WT and *OxCM* plants under Xoo stress, as the CM activity enhances Phe and Tyr biosynthesis. Our results showed that Phe was significantly increased 83%, 62% and 52% after 6, 12 and 24 h respectively in *OxCM* infected plants compared with WT infected plants (Fig. 3C). On the other hand, Tyr accumulation was the same in both the WT and transgenic plants after 1 h post-infection, but the accumulation was more reduced in WT as compared to *OxCM* plants with the passage of time (Fig. 3D). These results suggested that, during BLB infection, *OsCM* expression upregulates Phe biosynthesis while down regulating Tyr biosynthesis.

### *OsCM* regulates aromatic amino acid biosynthesis genes under stressed conditions

**Figure 4:**
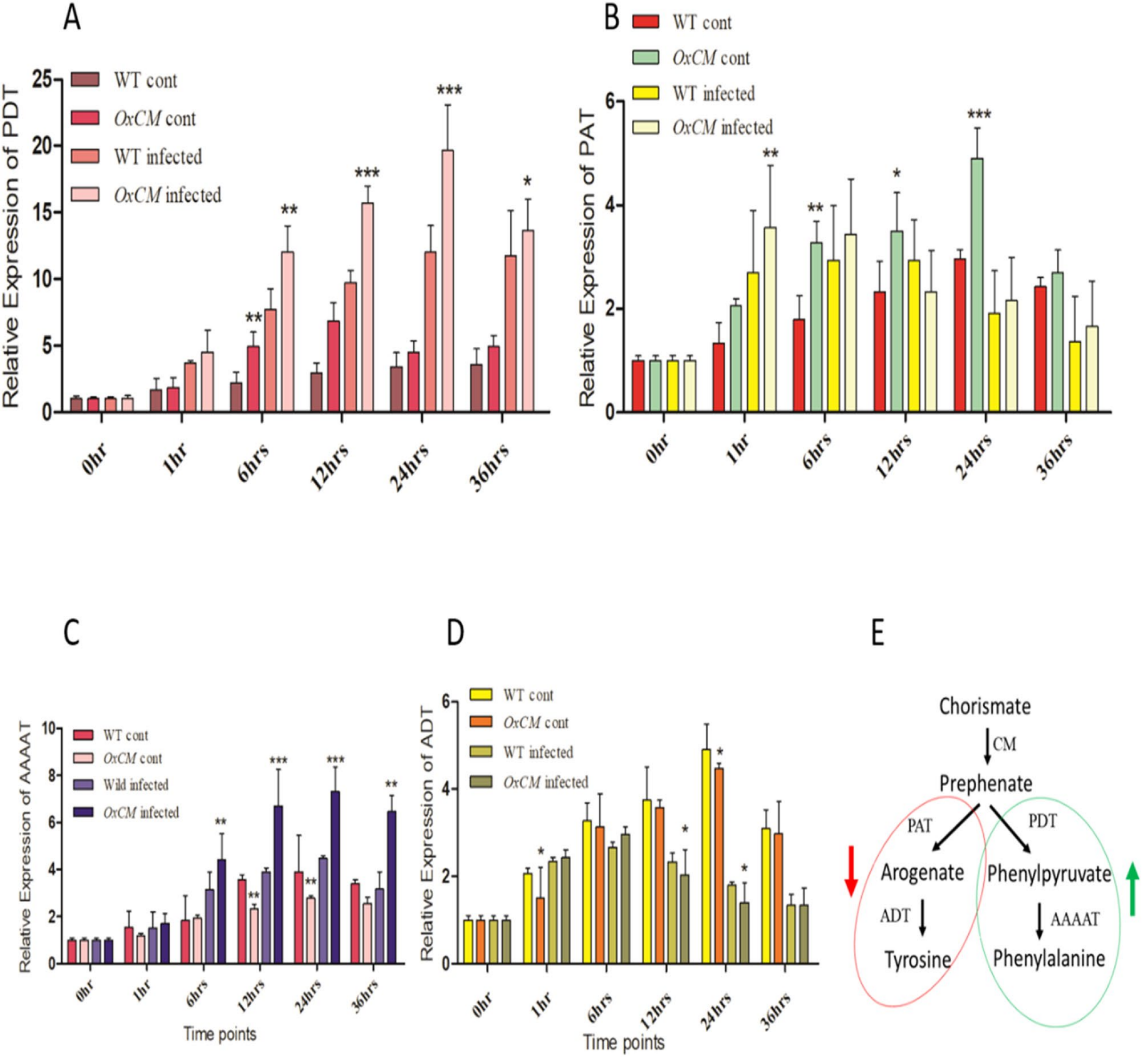
Regulation of downstream genes by overexpression of chorismate mutase. Bars represent mean ± standard deviation, while asterisks indicate a significant difference (*p *˂ 0 .05, two-way ANOVA, Bonferroni post-test). 0 h, 1 h, 6 h, 12 h, 24 h, and 36 h represent time points at which data were obtained (in hours). WT cont refers to the WT control, *OxCM* cont is the CM-overexpressing control line, wild infected is the WT infected with BLB, and *OxCM* infected is the CM-overexpressing line infected with BLB. (**A**) *PDT* is prephenate dehydratase, (**B**) *PAT* is prephenate aminotransferase, (**C**) *AAAAT* is the aromatic amino acid aminotransferase, and (**D**) is the arogenate dehydratase gene relative expression among the WT and *OsCM*-overexpressing infected and control plants. (**E**) A schematic representation of the related gene expression. A downward arrow indicates down regulation of *PAT* and *ADT*, while an upward arrow indicates up regulation of *PDT* and *AAAAT* genes at the same time.

To shed light on the genes which are involved in AAAs in the WT and *OxCM* plants under Xoo stress, we compared the transcript abundance of prephenate dehydratase (*PDT*), prephenate aminotransferase (*PAT*), aromatic amino acid aminotransferase (*AAAAT*), and arogenate dehydratase (*ADT*) genes, which are involved in AAA biosynthesis, by quantitative PCR (Fig. 4). We observed different expression level of these genes in WT and *OxCM* plants in Xoo-infected as well as uninfected plants. The relative expression of the *PDT* gene was significantly increased in *OxCM* infected plants compared with that in WT infected plants until 24 h and was then reduced at 36 h (Fig. 4A). Meanwhile, the expression of *PDT* was also increased in *OxCM* uninfected plants compared with that in WT uninfected plants, which suggested that overexpression of the *OsCM* gene also activates the *PDT* gene involved in the catalysis of prephenate into phenylpyruvate. Unlike *PDT*, *PAT* was initially (1 h) upregulated but was gradually down regulated in both *OxCM* infected plants and WT infected plants; however, the expression level remained higher in *OxCM* plants than in WT throughout 36 h (Fig. 4B). However, *PAT* expression was significantly higher in *OsCM*-overexpressing uninfected plants than in WT uninfected ones. These results suggested that induction of the PAT gene decreased in response to Xoo infection in both WT and *OxCM* plants, while under normal conditions, *OxCM* plants exhibit increased *PAT* expression compared with WT plants. We assumed that both *PDT* and *PAT* act antagonistically during Xoo infection. Similarly, *AAAAT*, which is involved in the regulation of phenylpyruvate conversion into Phe, showed an expression pattern similar to *PDT*. *AAAAT* was significantly expressed in *OxCM* infected plants compared with that in WT infected plants (Fig. 4C). However, the expression was reduced in *OxCM* uninfected plants compared with that in WT uninfected plants. Unlike *AAAAT*, *ADT* was expressed in *OxCM* infected plants compared with that in WT infected plants until 6 h after the inoculation of Xoo, but was then significantly reduced (Fig. 4D). On the other hand, similar to *PAT*, ADT was also upregulated in WT and *OxCM* uninfected plants, but the expression of *OxCM* was lower than in WT plants. Figure 4E shows a graphical representation of the effects of *OsCM-*overexpression on all of the four genes (*PDT*, *PAT*, *AAAAT*, and *ADT*) under stress conditions. It was assumed that, under stress conditions, the overexpression of *OsCM* down regulates the genes involved in the Tyr biosynthesis pathway while up regulating the genes involved in the Phe biosynthesis pathway.

### JA and SA cross-talk in *OxCM* and WT plants under pathogen infection

**Figure 5:**
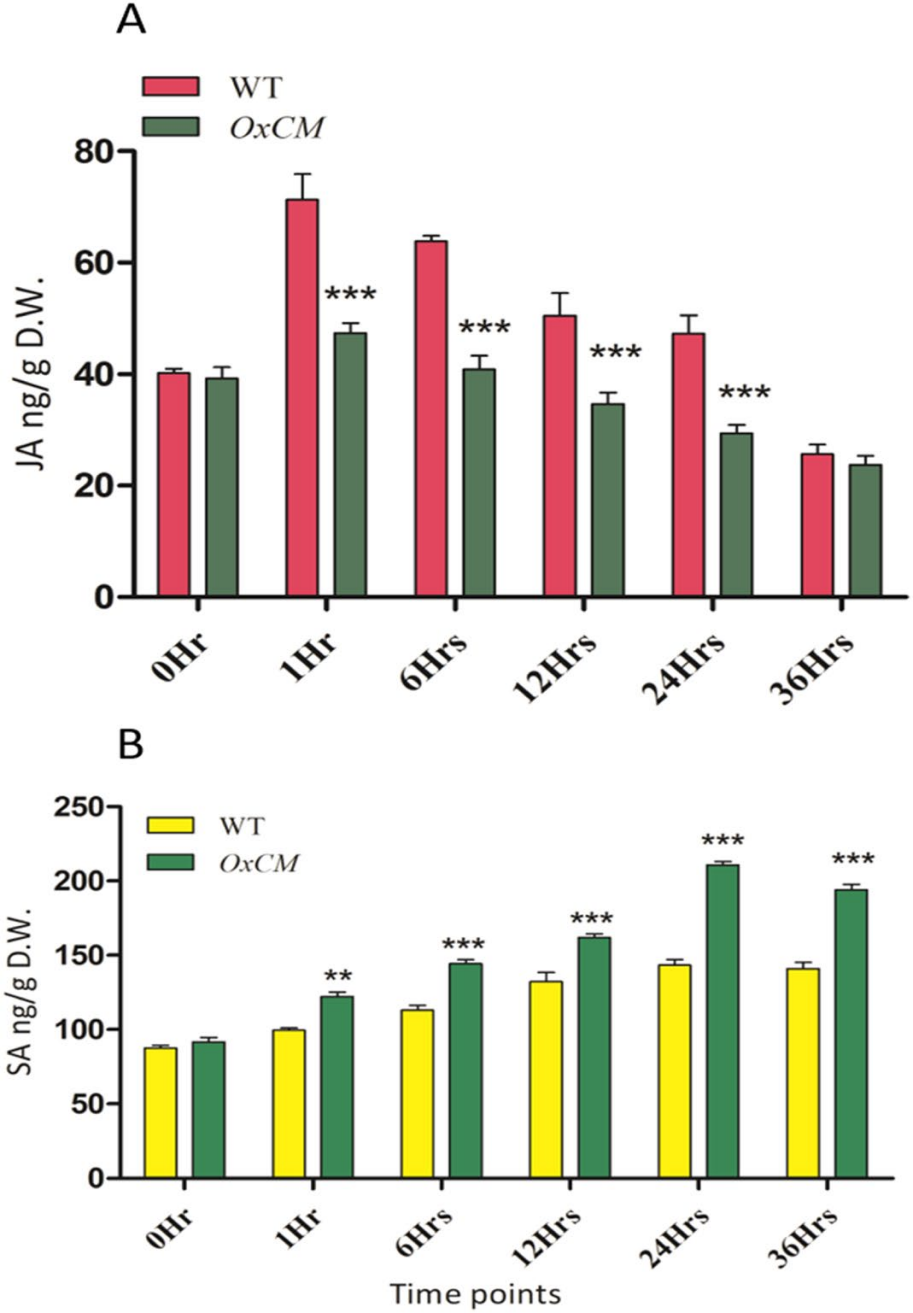
JA and SA accumulation in pathogen response. Bars represent mean ± standard deviation, while asterisks indicate a significant difference (*p *˂ 0.05, two-way ANOVA, Bonferroni post-test). 0 h, 1 h, 6 h, 12 h, 24 h, and 36 h represent time points at which data were obtained (in h). WT refers to WT infected and *OxCM* is the CM-overexpressing line infected with BLB. (**A**) JA accumulation and (**B**) SA accumulation in WT and *OxCM* infected plants.

To further verify the efficiency by which *OxCM* confers tolerance to Xoo infection with respect to that of WT plants, we evaluated the SA and JA accumulation in both types of plant under Xoo infection at six time points within 0–36 h. The results demonstrated that JA was significantly reduced in *OxCM* plants compared with that in WT plants (Fig. 5A). In the first hour of infection, the accumulation was increased in both types of plant, but later on it was consistently reduced. In the first hour of infection, about 70 ng/g D.W accumulated in WT plants, which was reduced to 30 ng/g D.W. which was 75%, after 36 h. Likewise, in the first hour of infection, 44 ng/g D.W. JA accumulated, which was reduced to 23 ng/g D.W. which was 47%, after 36 h. In contrast to JA, SA was significantly and continually enhanced in *OxCM* plants with increasing infection time, compared with that of WT plants (Fig. 5B). SA accumulated from 1 to 24 h in the range of 125–210 ng/g D.W. which was 68% however, from 36 h of infection, the SA level started to decrease. These results confirmed that JA and SA are regulated antagonistically.

### *OsCM* positively regulates the expression of disease resistance genes

**Figure 6:**
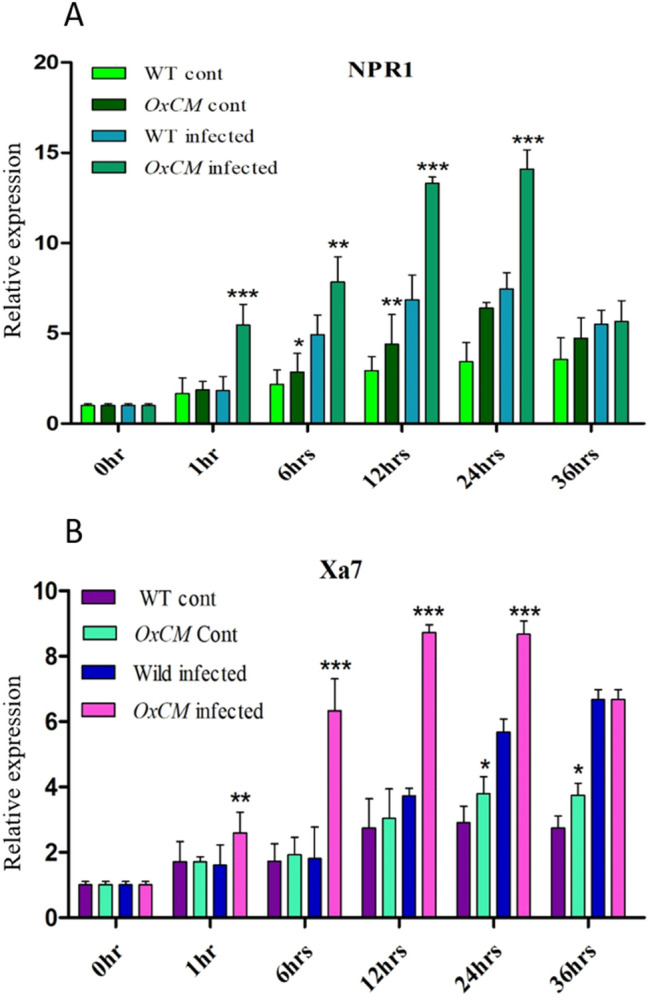
Expression of *NPR1* and *Xa7* under pathogen inoculation. Bars represent mean ± standard deviation, while asterisks indicate a significant difference (*p *˂ 0.05, two-way ANOVA, Bonferroni post-test). 0 h, 1 h, 6 h, 12 h, 24 h, and 36 h represent time points at which data were obtained (in hours), WT refers to WT control, *OxCM* cont is CM-overexpressing control line, wild infected is the WT infected with BLB, and *OxCM* infected is the CM-overexpressing line infected with BLB. (**A**, **B**) Relative expression of *NPR1* and *Xa7* genes, respectively, among the WT and *OsCM*-overexpressing control and infected lines.

It has been reported that *NPR1* is involved in Xoo resistance via regulation of the defense-related gene *Xa7*^25,26^. Thus, we examined the pattern of expression of *NPR1* and *Xa7* in the infected *OxCM* line with respect to infected WT plants. The results showed that *NPR1* was significantly induced in the *OxCM* infected plants compared with the level in WT infected plants and the expression increased with increasing infection time until 24 h (Fig. 6A). After 24 h of infection, the *NPR1* expression was reduced in infected and uninfected *OxCM* and WT plants. However, the *NPR1* gene expression was also higher in *OxCM* uninfected plants than in WT uninfected plants. Likewise, the *Xa7* gene also showed the same pattern of expression as *NPR1*. The *Xa7* expression was also higher in *OxCM* infected plants than in WT infected ones (Fig. 6B). The expression gradually increased with increasing infection time until 24 h. The results suggest that overexpression of *OsCM* in rice plants increases BLB tolerance in terms of regulating the *NPR1* gene, which in turn regulates the defense-related gene *Xa7*.

### *OsCM* enhances lignin accumulation and amino acid content upon BLB stress

**Figure 7:**
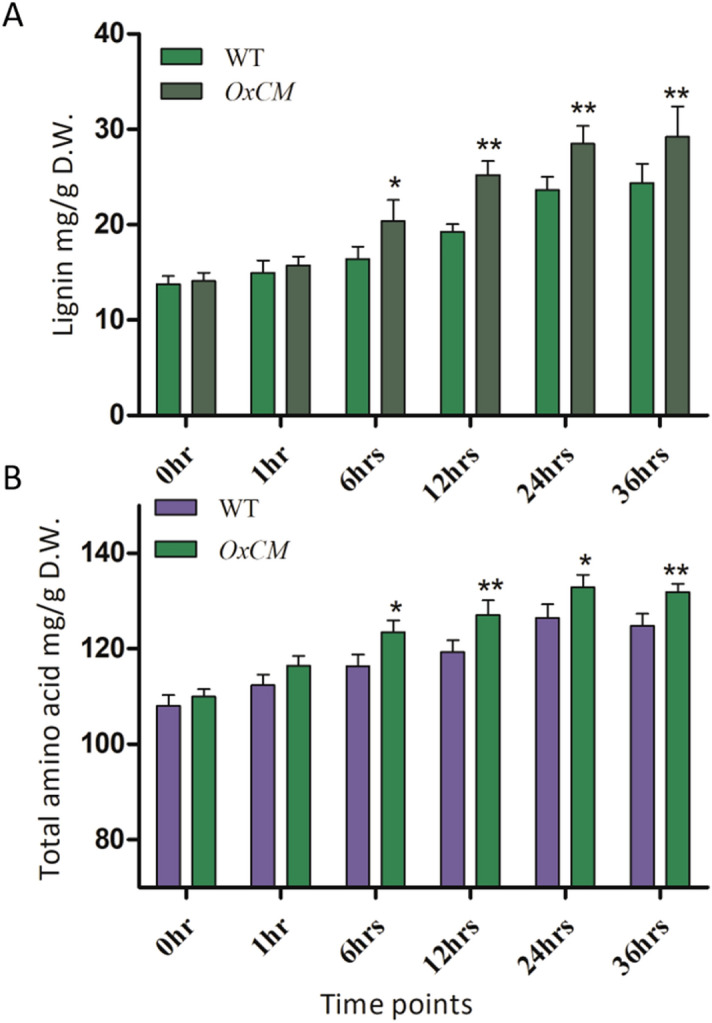
Lignin and amino acid accumulation upon pathogen stress. Bars represent mean ± standard deviation, while asterisks indicate a significant difference (*p *˂ 0.05, two-way ANOVA, Bonferroni post-test). 0 h, 1 h, 6 h, 12 h, 24 h, and 36 h represent time points at which data were obtained (in h). WT refers to WT infected and *OxCM* is the CM-overexpressing line infected with BLB. (**A**) Lignin accumulation and (**B**) total amino acid accumulation in WT and *OxCM* infected plants.

We studied lignin and total amino acid contents against Xoo stress in *OxCM* and WT plants, as lignin exhibits regulatory functions under biotic as well as abiotic stresses, while amino acids are involved in the biosynthesis of various secondary metabolites, which are directly involved in biotic stresses^27,28^. The results showed that more lignin and total amino acids were accumulated in the *OxCM* infected plants as compared to WT infected plants (Fig. 7A,B). Continuous increases of lignin and amino acids were found upon continued infection. Lignin was increased 115% from 13 mg/g D.W. to 28 mg/g D.W. from 0 to 36 h of infection in *OxCM* plants. Similarly, the concentration of amino acids in *OxCM* infected plants increased 21% from 107 to 130 mg/g D.W. over 36 h of infection. However, significance differences in both lignin and amino acids appeared after 6 h of infection. These results show that Xoo infection regulates lignin and total amino acids in rice plants.

## Discussion

AAAs such as Phe and Tyr are central molecules in plant metabolism and synthesized in the shikimate pathway from the common precursor chorismate. The shikimate pathway connects primary metabolites to secondary metabolites via chorismate being the end product of shikimate, which initiates the synthesis of AAAs that in turn enhances secondary metabolites^3^. Phe and Tyr induces cinnamic acid which is the precursors of phenylpropanoid^9^. Plants have adopted several defense signaling pathways to mitigate severe environmental conditions and pathogenic attack, such as by altering secondary metabolites^29^. The modulation of secondary metabolites like phenylpropanoids against biotic and abiotic stresses is a clue to the involvement in stress tolerance^30^. A wide range of phenolic compounds are produced from phenylpropanoids such as flavonoids, isoflavonoids, anthocyanins, plant hormones, phytoalexins, and lignins^31,32^.

In current study, *OsCM* gene was functionally evaluated by generating an overexpressing line using *Agrobacterium*-mediated transformation in rice plants. The innate immunity of plants was altered upon infection of BLB and the suppression and induction of *OsCM* activity were confirmed by biological assays. The phenotypic evaluation of lesion length demonstrated that *OxCM* plants were highly tolerant of BLB stress. Evaluation of the two AAAs, Phe and Tyr, showed that they were adversely altered during BLB stress, which suggested that they are participants in pathogen tolerance. The results showed that Phe accumulation was increased in the *OxCM* line, while Tyr was decreased in Xoo after inoculation (Fig. 3C,D). We predicted that the alterations of Phe and Tyr were due to induction of the *OsCM* gene because the *OsCM* gene was significantly induced in the *OxCM* line at the transcriptomic and proteomic levels (Fig. 3A,B), which enhanced the accumulation of Phe in the transgenic line. Phe is considered to be the chief AAA accumulated in plants under normal conditions, being at a level higher than Tyr and Trp, and absorbing about 30% of photosynthetic carbon to produce phenylpropanoids^33^. We assumed that the induction of Phe under BLB stress in *OxCM* plants, enhanced the accumulation of downstream metabolites, which increased Xoo tolerance. A previous study showed that induction of the derivatives of Phe and Tyr (phenylpropanoids) under stress conditions was enhanced due to up regulation of the Phe catabolic enzyme, *PAL*, which uses Phe as a substrate^34^. Supported by the high accumulation of Phe in *OxCM* infected plants, the results showed that the genes downstream of *OsCM* such as *PDT* and *AAAAT* were positively regulated, whereas the genes responsible for Tyr biosynthesis such as *PAT* and *ADT* were negatively regulated (Fig. 4). The fold change of Phe was much greater than that of Tyr, which shows that Phe is highly involved in the biosynthesis of phenylpropanoids in response to pathogenic attack. A previous study confirmed that an increasing Phe level in petunia flowers increases the production of phenylpropanoids^35^. The signaling of a reduction in Tyr corresponding to Phe in BLB stress is not well known, but it was predicted that Tyr is not efficiently involved in stress mitigation compared with Phe.

Plant hormones are dynamic regulators of plant responses to environmental pressure by participating external stimuli with complex monitoring systems. SA is an important part of plant defense and is well known for its defensive role in plant defense system against pathogens^36^. SA has a vital role in rice defense system in response to Xoo, and enhances both basal defense and the hypersensitive response during Xoo infection in rice^20^. The SA signaling pathway might be involved in the regulation of glucosinolate, which is hydrolyzed by SA to various degradation products that play significant part in interactions with pathogens^37^. The current study investigated the regular increase of SA in rice plants in response to continued Xoo infection, and the SA accumulation was significantly higher in *OxCM* plants than in WT. This investigation proved that *OsCM* is significantly involved in the biosynthesis of SA. In most plants, SA can be synthesized by two key regulatory enzymes, *PAL* and *ICS*, whereas *PAL* uses phenylalanine and *ICS* uses chorismate as a substrate^38^. Although *ICS* is involved in SA accumulation in response to some plant stresses^11,39^, the pathogen and other stress-induced SA is mostly derived from the *PAL*-catalyzed pathway^40^. A previous study showed that *Arabidopsis PAL* mutant accumulated more SA than *ICS* mutant, which suggested that, in *Arabidopsis*, *ICS* is the key contributor to pathogen-induced SA biosynthesis^11^. However, these clarifications are based on independent analysis of either the *PAL* or the *ICS*-catalyzed pathway, and the comparative contributions of *PAL* vs. *ICS* to SA biosynthesis and pathogen defense have not been instantaneously assessed for any plant system. Although the *PAL* and *ICS* pathways are equally significant for SA biosynthesis, suppression of either pathway is enough to compromise SA biosynthesis in response to pathogenic attack. In the present study, we did not evaluate the relative expression of *ICS*, but the higher accumulation of *Phe* and SA suggested that *PAL* is sufficiently involved in the biosynthesis of SA in response to BLB stress. A previous study showed that SA regulation in response to stress may be feedback-regulated by the metabolites of biochemical pathways. This inference was supported by the feedback-regulation of CM by phenylalanine and feedback allosteric regulation assessed in amino acid biosynthesis^41,42^. Notably, the bacterial pathogen infection increased the expression level of the key leaf-expressed *PAL* isoform instead of *ICS* in soybean. Another previous study suggested that either *PAL* or *ICS* knockout eliminated pathogen-induced production of SA in soybean, which implied that the expression of *ICS* is not a requirement for SA biosynthesis^38^. It is possible that enhanced *PAL* induction as related to its role as a chief regulator of the phenylpropanoid pathway, which is well known to participate in defense^43–45^. Similar to SA, JA also has a main part in mitigating pathogen-related stress. However, SA and JA signaling pathways were reported to interact antagonistically against biotic stress^46^. Unlike SA, the JA accumulated in *OxCM* plants was efficiently reduced with increasing Xoo infection time, compared with that in the WT (Fig. 5A). Studies have shown that SA facilitates the down regulation of JA-responsive genes like lipoxygenase 2 (*LOX2*), vegetative storage protein, and *PDF1.2*^24^. Pathogenic infection manipulates the defense-related regulatory network in plants in terms of phytohormone accumulation, which causes hormonal imbalance and activates untimely defense responses^47^. Previous studies showed that the synthesis of coronatine-1 JA-Ile mimic by *Pseudomonas syringae pv.* tomato (Pst) bacteria altered the stimulation of JA-dependent defense responses, leading to the inhibition of SA-dependent defense responses^48,49^. Plants usually resist a diverse range of attackers in the natural environment via regulating complex defense mechanisms to modulate effective defense responses against pathogens. However, the mechanism by which plants prioritize one response over another is unknown. This interpretation shows that not all pathogens activate JA signaling in plants, such as *Bemisia tabaci* enhancing SA and suppressing JA accumulation in *Arabidopsis*^50^, similar to our findings. This means that neither SA nor JA is more important, but both hormones are equally important for pathogen resistance. Positive or negative cross-talk between SA and JA may be induced depending on the pathogen^51^.

This study was extended to focus on the relative expression levels of *NPR1* and *Xa7* genes. *NPR1* is one of the essential regulatory tools of SA signaling, which interacts with TGA transcription factors that are responsible for the activation of SA-responsive *PR* genes^52^. *NPR1* in rice enhances tolerance to rice bacterial blight (*Xanthomonas oryzae* pv. oryzae) and rice blast fungus (*Magnaporthe grisea*), and also activates *PR* gene induction and regulates SAR^22^. *Xa7* is a *PR* gene that enhances Xoo resistance in rice plants at high temperature, whereas resistance change due to other *R* genes is usually suppressed^53^. *Xa7* regulates hypersensitivity and localized host cell death to control pathogenic infection in the plants^54^. It was previously shown that *Xa7* expression suppressed the expression of ABA-related genes, which signal antagonistically with SA^55^. Our results suggested that SA and *Xa7* interact with each other and are positively regulated in response to pathogens. Furthermore, our study revealed that the expression of *NPR1* and *Xa7* was highly induced in the *OxCM* line under Xoo stress, which was consistent with the higher SA accumulation (Fig. 6). A previous investigation evaluated that the enhancement of SA accumulation in pathogen-infected tissues resulted in enhancement of the PR gene expression that modulate the resistance of a broad range of pathogens^46^. *NPR1* plays an essential part in SA–JA cross-talk because the *NPR1 Arabidopsis* plants were compromised in terms of the SA-mediated suppression of JA-responsive gene expression^56^. Various WRKY transcription factors downstream of *NPR1* play key roles in altering SA-dependent defense responses in plants^21^. SA’s association with heme-containing enzymes, such as catalase, result in adequate redox stress to initiate the release of monomers of *NPR1* and their entry into the nucleus^57^.

Lignin and amino acids are essential modulators of stress responses in plants produced via the shikimate pathway^58^, associated with the *PAL* pathway. Along with the accumulation of SA, *PAL* activity also offers a precursor (cinnamic acid) for lignin biosynthesis in response to pathogen infection^59^. We investigated that lignin quantification in response to Xoo infection was higher in *OxCM* rice plants than in the WT. It was assumed that overexpression of the *OsCM* gene was involved in the regulation of lignin via the alteration of *PAL* activity. In *PAL-*knockdown *Brachypodium* plants, lignin accumulation was decreased up to 40%, which enhanced pathogen susceptibility^60^. Lignin accumulation in *Arabidopsis PAL1*, *PAL2*, *PAL3*, and *PAL4* mutants was decreased from 20 to 25% compared with that in WT plants, while the same plants also showed a reduced level of SA and enhanced pathogen susceptibility^61^. Although lignification enhances the toughness of the cell wall in response to pathogenic attack, the free radical-mediated polymerization of lignin precursors in intercellular spaces might also lignify pathogenic structures. Lignin hydrophobicity enables solute transformation in the vascular tissues and reduces water loss during evapo-transpirative processes. Similarly, total amino acid accumulation was enhanced in *OxCM* infected plants compared with that in WT plants because the higher accumulation of amino acids is essential for plants to respond to stress in term of ROS scavenging behavior, as well as potential regulatory and signaling molecules^62,63^. The higher accumulation of total amino acids in Xoo-infected *OxCM* plants suggested that the *OsCM* gene of the shikimate pathway is significantly involved in the accumulation of total amino acids in response to pathogenic attack.

## Materials and methods

### Generation of *OsCM* transgenic rice line

Rice plants (Cheongcheong) were selected for transformation and further study. The seeds were sterilized with 3% hypochlorite for 10 min and then incubated at 32 °C in water until they started to germinate, with their water changed every day. The germinated seeds were grown in a greenhouse and the samples were collected after 3 weeks for total RNA isolation. Total RNA was isolated using RNeasy Plant Mini Kit from Qiagen and the open reading frame (ORF) of *OsCM* (MH752192) was amplified using gene primers. The *OsCM* was cloned into the cloning vector pENTR/D-TOPO (pENTR Directional TOPO cloning kit; Invitrogen) and then into the PSB11 expression vector using the Gateway cloning system (Gateway LR Clonase enzyme mix kit; Invitrogen). The entry clone was first transferred into DH5α *E. coli* and then transferred into *Agrobacterium* cells LBA4404 (Takara) via the heat shock method and spread on hygromycin-containing LB medium. Both inserts were confirmed through double digestion using Not1 and Asc1 and BamH1 and Xho1, respectively. *CM*-overexpressing (*OxCM*) rice lines were developed through a callus culture technique. Seeds of good quality were dehulled, sterilized with 3% hypochlorite for 10 min, washed three times, sterilized again with 70% ethanol for 5 min, washed again with ddH_2_O, and dried in laminar flow. Dried seeds were inoculated in callus induction medium at 10–15 seeds per plate and placed in the dark for 12 days. The induced callus was further pre-cultured on callus induction medium for 3 days under dark conditions. At the same time, the *OsCM*-inserted *Agrobacterium* cells were grown on selection medium for transformation into callus. *Agrobacterium* cells were pelleted and resuspended in MS medium fortified with acetosyringone and the callus was immersed in the suspension for 30 min with continuous shaking. After incubation in *Agrobacterium* cells, the callus was dried for 30 min on sterilized filter paper and then inoculated into co-cultivation medium for 3 days in the dark. The excessive growth of *Agrobacterium* in the callus was controlled by washing three times with 500 mg/l carbenicillin, dried for 30 min, and again inoculated into the first selection medium containing 50 mg/l hygromycin. The callus was inoculated three times into selection medium under light conditions (16/8 h photoperiod). After three periods of selection, the callus was transferred into regeneration medium for 10 days in the dark. In the second phase, the callus was transferred to new identical medium and placed under light conditions until the plantlets developed; in the third phase, the plantlets were put in a test tube on the same medium to develop roots. After 20 days in a test tube, the plants developed appropriately and were transferred to soil in pots.

### Inoculation of BLB and lesion length measurement

Wild-type and *OxCM* plants were grown in a greenhouse, following the described method. The K3a strain of *Xanthomonas oryzae* (Xoo) was inoculated into wild-type as well as *OxCM* plants using the leaf clipping method^64^. The experiment was conducted in two sets: one set of wild-type and *OxCM* plants were infected with Xoo, while the second set remained uninfected and was used as a control. The samples were collected in triplicate 0, 1, 6, 12, 24, and 36 h after the inoculation of Xoo for further analysis. However, lesion length was measured after each week until 5 weeks after Xoo infection.

### RNA isolation and quantitative RT-PCR analysis

Total RNA was isolated from five leaves in triplicate and cDNA was synthesized using the qPCRBIO cDNA Synthesis Kit from PCRBIOSYSTEMS. Quantitative real-time RT-PCR (qRT-PCR) was performed using qPCRBIO SYBR Green Kit from PCRBIOSYSTEM, using cDNAs as templates and gene-specific primers. To normalize the level of relative expression of each gene, actin was used for each reaction and the expression level was calculated in wild-type plants relative to that in *OxCM* infected ones. The reaction was performed in a 20 µl volume containing 7 µl of ddH_2_O, 1 µl of primer, 10 µl of SYBR green, and 1 µl of cDNA, and was repeated in triplicate.

### Western blot analysis

To check protein expression in the transgenic line as well as in the wild-type, western blotting was performed by a previously reported optimized method^65^ with slight modifications. Proteins of the *OxCM* line and the wild-type treated with BLB were collected at three time points: 2, 6, and 12 h after BLB inoculation. Total protein was isolated with 10 ml of TCA/acetone [10% trichloroacetic acid (TCA); 0.07% β-ME in acetone P.A.], by a previously reported method^66^. Equal amounts of protein were boiled for 5 min, separated by10% SDS-PAGE at 100 V for 150 min, and then transferred to an NC membrane (Whatman Japan) by a semi-dry method running for 90 min at 19 V using Trans-Blot DS semi-dry transfer cell (Bio-Rad). The membrane was blotted in TBST (0.1% Tween 20 in TBS) and 5% non-fat dry milk (w/v) for 2 h at room temperature. Proteins were further blotted with primary rabbit anti-CM synthase antibodies in 5% non-fat dry milk (w/v) in TBST overnight at 4 °C and subjected to three rinses for 10 min each in TBST solution. The membrane was then incubated with Gt anti-Ms IgG (H + L) secondary antibody (Invitrogen, USA) at a dilution of 1:1000 for 2 h at room temperature and rinsed three times for 10 min each in TBST solution. The blot was developed by Amersham ECL (GE Healthcare, UK) and protein bands were exposed on X-ray film.

### Quantification of endogenous SA and JA hormones

To assess SA and JA accumulation in wild-type and *OxCM* plants in response to Xoo stress, we analyzed both of these hormones. Leaves of the plants were collected in liquid nitrogen after each time point and stored at − 80 °C. For SA analysis, frozen leaves were lyophilized and crushed into a fine powder in liquid nitrogen using a modified version of a previously reported protocol^67^. The lyophilized powder of each sample (0.3 g) was extracted with 90% ethanol and 100% methanol and centrifuged for 20 min at 1000 rpm. The supernatant was collected and methanol was dried in a vacuum centrifuge and again resuspended in 5% trichloroacetic acid (3 ml). The supernatant was further mixed with ethyl acetate/cyclopentane/isopropanol (49.5:49.5:1, v/v), and the uppermost organic layer was collected in a 4 ml vial and then dried with nitrogen gas. The extracted SA was analyzed by HPLC, with quantification through fluorescence detection. For JA analysis, freeze-dried leaves (0.2 g) were homogenized with liquid nitrogen and JA was extracted with acetone and 50 mM citric acid (70:30, v/v), following a previously reported protocol^68,69^. An internal standard, [9,10-2H2]-9,10-dihydro-JA (20 ng), was also added to the suspension. The extract was kept at low temperature overnight to evaporate highly volatile organic solvents and retain the less volatile fatty acids. The remaining solution was filtered and then extracted with 10 ml of diethyl ether three times. The extract was further loaded onto a solid-phase extraction cartridge (500 mg of sorbent, aminopropyl) and the cartridges were cleaned with 7.0 ml of 2-propanol and trichloromethane (1:2, v/v). The JA and related standard were eluted with 10 ml of diethyl ether and acetic acid (98:2, v/v). The residue of solvents after evaporation was esterified with diazomethane and analyzed by GS-MS (6890 N network GC system and the 5973 network mass-selective detector; Agilent Technologies, Palo Alto, CA, USA) in the selected ion mode. The ion fragment was monitored at m/z = 83 amu, consistent with the base peaks of JA and [9,10-2H2]-9,10-dihydro-JA; the JA was quantified from the peak areas corresponding to the respective standards.

### Measurement of lignin content

Lignin content was assessed following a modified version of a previously reported method^58^. Samples weighing 0.6 g stored at − 80 °C were ground in liquid nitrogen and washed five times with 95% ethanol to eliminate soluble metabolites. The homogenate was further washed with acetone and dried in an oven. The dried sample was further disrupted in acetic acid using an ultrasonic machine and centrifuged at 3000 × *g* for 5 min. The remaining pellet was resuspended in 25% acetyl bromide and again centrifuged at 3000 × *g* for 5 min. The samples were mixed with a mixture of acetic acid and acetyl bromide (4:1, v/v) and heated at 70 °C for 2 h. The samples were cooled at room temperature and transferred to 50 ml of 2 M sodium hydroxide, 1.5 ml of acetic acid, and 7.5 M hydroxylamine hydrochloride. The volume of each sample was equalized with acetic acid. The supernatant’s absorbance was measured with a spectrophotometer at 280 nm (UV-2450; Shimadzu, Japan).

### Statistical analysis

All experiments of each section were performed in triplicate, and the data collected from each replicate were pooled together. The data were analyzed using two-way ANOVA followed by Bonferroni post hoc test (significant difference: *p *˂ 0.05). A completely randomized design was used to compare the mean values of different treatments. The data were graphically presented and the statistical analyses were performed using GraphPad Prism software (version 5.01; GraphPad, San Diego, CA, USA).

## Conclusion

This study has provided evidence that the overexpression of *OsCM* potentially accelerated resistance to BLB stress in rice. The results revealed that the expression of *OsCM* significantly induced the accumulation of aromatic amino acids, as well as regulation of the PAL pathway and pathways downstream from AAAs. This induction led to the production of specialized metabolites such as lignin, the accumulation of hormones such as SA and JA, and alteration of some specialized amino acids that protect cells from oxidative damage. Here, we found that AAAs such as Phe and Tyr are not only essential for protein synthesis but also serve as precursors of the PAL pathway, which is responsible for the synthesis of a wide range of secondary metabolites. Increased PAL pathway function by the overexpression of *OsCM* has a positive impact on BLB resistance. Owing to the high accumulation of Phe and up regulation of the genes responsible for the synthesis of Phe compared with that for Tyr in the transgenic line, it was found that Phe is strongly associated with pathogen-related stress compared with Tyr. SA is an essential regulator of plant defense by inducing the expression of defense-related genes. The SA pathway was upregulated while JA was down regulated in infected transgenic plants, which suggested that SA accumulation enhances *NPR1* and *Xa7* expression in response to pathogen-related stress. The positive responses of lignin and total amino acid accumulation in response to pathogen-related stress in the *OsCM*-overexpressing line also indicated that lignin and amino acids are essential parts of plant defense. These results show that AAAs, the PAL pathway, hormones, and pathogen-resistant genes function cooperatively in response to BLB stress in rice.

## Supplementary information

Supplementary Figures.
