# Supplementary material for: Overexpression of OsCM alleviates BLB stress via phytohormonal accumulation and transcriptional modulation of defense-related genes in Oryza sativa
Source: Sci Rep. 2020 Nov 11;10:19520. doi: 10.1038/s41598-020-76675-1 (PMC7658211; doi:10.1038/s41598-020-76675-1)
Supplement: Supplementary file 1 — Supplementary Figures. [file 41598_2020_76675_MOESM1_ESM.docx]

**Overexpression of *OsCM* alleviates BLB stress via phytohormonal accumulation and** **transcriptional modulation of defense-related genes in *Oryza sativa***

Rahmatullah Jan^1^, Muhammad Aqil Khan^1^, Sajjad Asaf^2^, In-Jung Lee^1^, Jong-Sup Bae^3*^, Kyung-Min Kim^1*^

^1^Division of Plant Biosciences, School of Applied Biosciences, College of Agriculture & Life Science, Kyungpook National University, 80 Dahak-ro, Buk-gu, Daegu, 41566, Republic of Korea

^2^Natural and Medical Science Research Center, University of Nizwa 616, Oman

^3^College of Pharmacy, CMRI, Research Institute of Pharmaceutical Sciences, BK21 Plus KNU Multi-Omics based Creative Drug Research Team, Kyungpook National University, Daegu 41566, Republic of Korea

^*^Corresponding authors: Jong-Sup Bae, baejs@knu.ac.kr; Kyung-Min Kim, kkm@knu.ac.kr

Phone: +82-52-950-8570; +82-53-950-5711, Fax; +82=53-950-8557; +82-53-958-6880


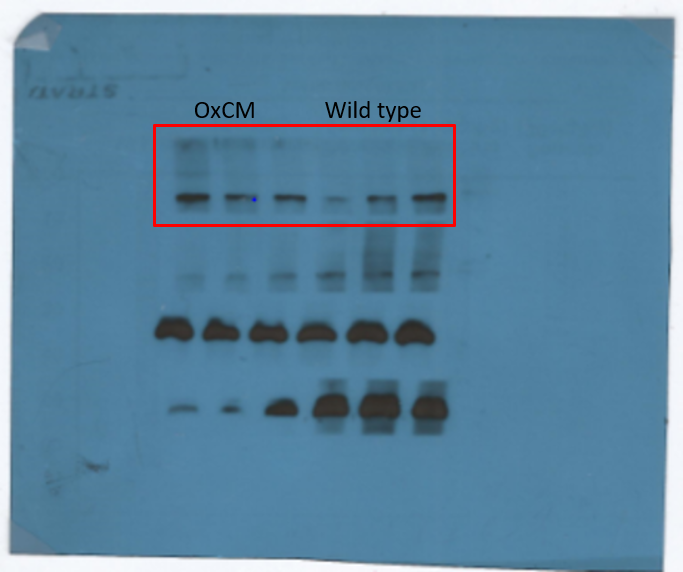


**Figure S1.** This is the original picture of figure 3B, unprocessed. However, this is also cropped because this gel was blotted together with other samples on one X-ray film and then we cropped accordingly.


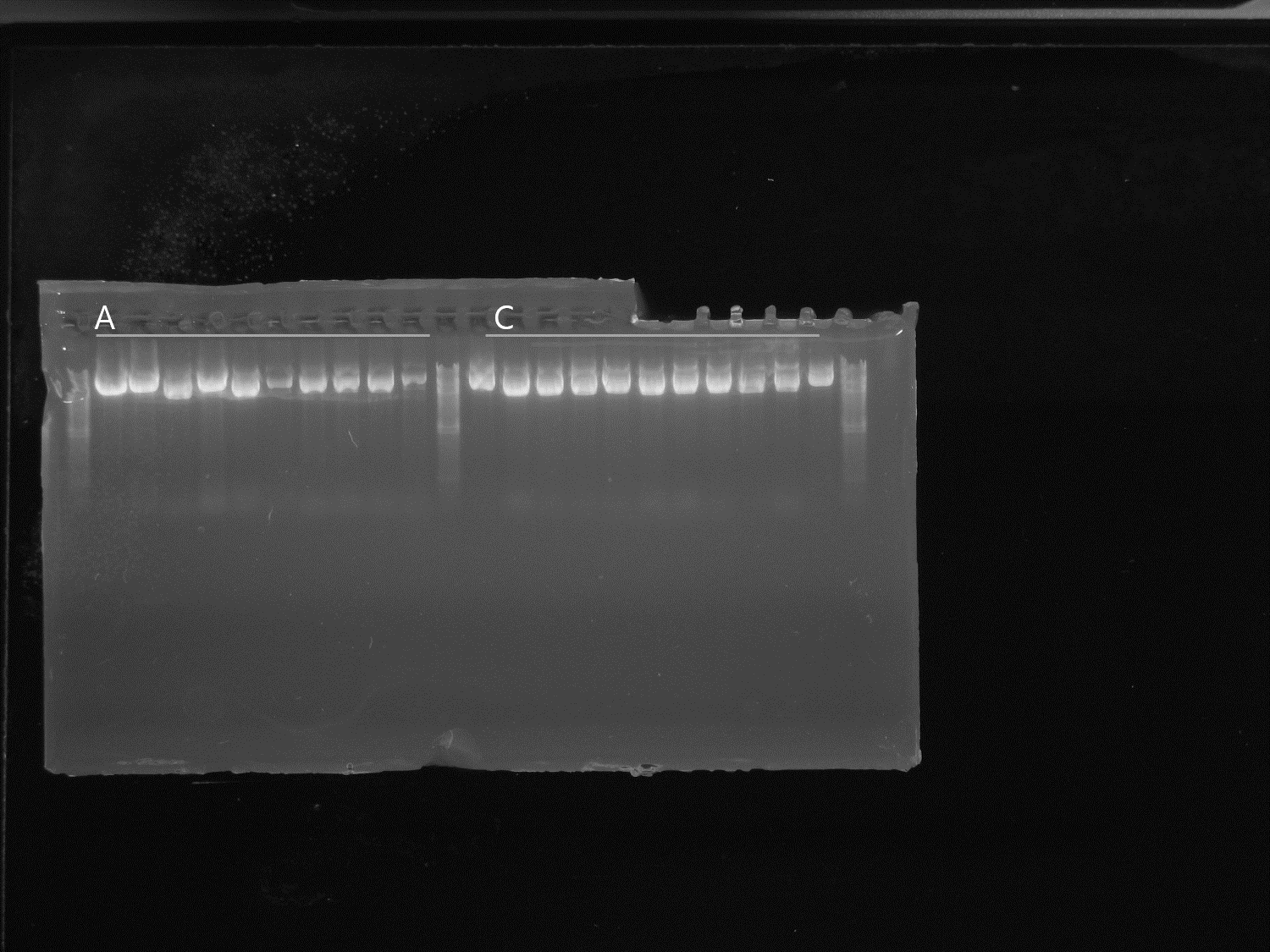


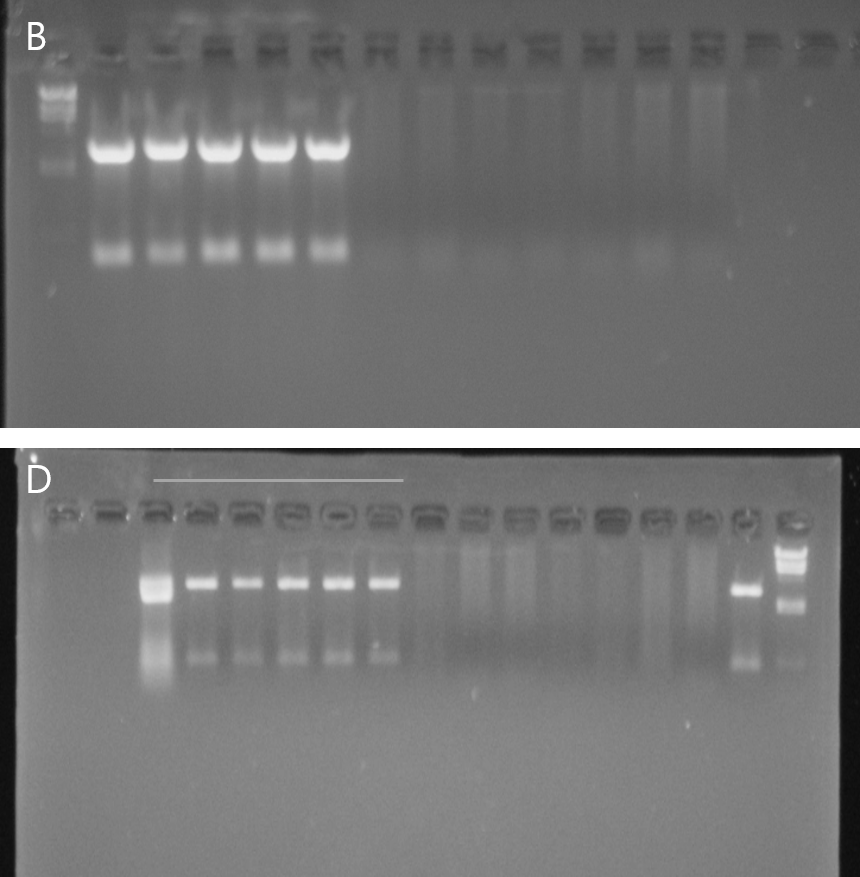


**Figure S2.** This is the original un-processed picture of figure 1 A-D.
